# Biological Models for Evaluating Hydrogel-Based Formulations in Wound Healing

**DOI:** 10.3390/gels11090705

**Published:** 2025-09-03

**Authors:** Ioana Baldea, Ioana Georgeta Grosu, Sahar Ghafury, Cristian Golat, Doriane Doubali, Ana-Maria Vestemean, Aris Nicolas Cedorge, Ilinca Florian, Michael Yiannoulatos, Muhammad Mudassir Wajahat, Lorenzo Raoul Silli, Thesseus Stavrou, Daniela Rodica Mitrea

**Affiliations:** 1 Department of Physiology, University of Medicine and Pharmacy, Clinicilor 1, 400006 Cluj-Napoca, Romania; baldeaioana@gmail.com (I.B.); cristigolat@gmail.com (C.G.); dododoubali@gmail.com (D.D.); amvestemean@gmail.com (A.-M.V.); ariscedorge2015@gmail.com (A.N.C.); ilincaflorian16@gmail.com (I.F.); mike1325gia@gmail.com (M.Y.); mmw.of2004@gmail.com (M.M.W.); lorenzo.silli@yahoo.fr (L.R.S.); thesseus.stavrou@gmail.com (T.S.); rdmitrea@yahoo.co.uk (D.R.M.); 2 National Institute for Research and Development of Isotopic and Molecular Technologies, Donat 67-103, 400293 Cluj-Napoca, Romania

**Keywords:** scratch assay, reconstructed human epithelium, full-thickness skin model, wound healing models, surgical wound model, burn wound model, hydrogel-based dressings, crosslinked hydrogels

## Abstract

Skin, the largest organ of the human body, serves as a critical physico-chemical barrier against environmental insults and plays essential roles in hydration, thermoregulation, immune defense, and metabolic functions. Wound healing is a complex, multistage biological process involving hemostasis, inflammation, proliferation, and remodeling. Hydrogels have emerged as a promising class of wound dressings due to their high moisture retention, biocompatibility, and ability to mimic the extracellular matrix, thereby supporting accelerated healing and controlled drug delivery. This review provides a comprehensive overview of current hydrogel types—classified by origin, crosslinking mechanisms, and responsiveness to stimuli—and evaluates their use in experimental research on in vitro, ex vivo, and in vivo wound healing models. Furthermore, clinical applications of hydrogels in wound therapy are discussed. Advances in semisynthetic and stimuli-responsive hydrogels, along with improved testing models, offer enhanced therapeutic potential and underscore the need for continued innovation to optimize wound care outcomes and alleviate healthcare burdens.

## 1. Introduction

Skin is the largest human body organ, providing the initial physico-chemical barrier against saprophytes and pathogens, radiation, such as ultraviolet irradiation, and chemical and mechanical injuries. Besides the barrier function, skin is an active part of many other homeostatic mechanisms. It maintains the body’s hydration state, thermoregulation, immune recognition and defense, synthesis of vitamin D precursors, and excretion of some catabolites and drugs [1].

Millions of skin injuries caused by physical or thermal agents occur annually, and if not properly treated, they can develop into chronic wounds. Wounds are defined as any damage or disruption of the body’s tissues, typically the epidermis. They can be classified into open (laceration) and closed (hematomas). According to the wound age, onset mechanism, and healing capacity, they can be acute or chronic [2].

The wound healing process is a physiological reaction immediately initiated after tissue injury. It is a dynamic and multistage biological response comprising four overlapping phases: hemostasis (immediate), inflammation (days 1–4), proliferation (days 4–21), and remodeling or maturation (day 21 to up to 2 years).

In the initial phase, characterized by loss of lymphatic fluid and/or blood, hemostasis is crucial, leading to the formation of a platelet plug and eventually a stable clot. These also lead to initiation of the inflammatory reaction triggered by serotonin and histamine released from thrombocytes and local leukocytes. Inside the fibrin network there are scattered neutrophils and leukocytes that decontaminate the wound and phagocytose bacteria and cellular debris. The wound contracts due to increased fibroblast and myofibroblast activity. By days five to seven, fibroblasts start collagen and glycosaminoglycans synthesis to form the core of the granulation tissue. Migrating cells, mainly keratinocytes from the outskirts of the plaque, move towards the wound and start re-epithelialization of the wound surface. This is sustained by the ongoing angiogenesis from the existing vessel network, where the endothelial cells proliferate and start migrating. Their proliferation leads to new vascular sprouts that replace and heal the wounded vascular network. However, the scar tissue does not bear the same strength as the initial tissue. In the maturation phase, the newly formed scar tissue is remodeled to reduce thickness and increase elasticity and strength as a response to mechanical stress. Also, pigmentogenesis is activated due to melanocyte activity and migration, particularly as a response to inflammation and ultraviolet exposure [2,3].

This intricate process can be disrupted by various physiological factors such as aging and infection or by pre-existing medical conditions like diabetes and obesity. This may lead to delayed healing or development of chronic wounds, often accompanied by complications including severe inflammation, infection, hemorrhage, and, in extreme cases, limb amputation [4,5].

To support optimal healing, appropriate wound management is essential, and the choice of dressing plays a central role. An ideal wound dressing should maintain a moist environment to promote cell migration and tissue regeneration, manage exudate to prevent maceration, permit gas exchange, and provide a barrier against microbial contamination. Furthermore, dressing must be sterile, non-toxic, nonallergenic, and comfortable, while also being easy to apply and remove without causing further tissue damage [6]. Traditional dressings such as gauze and cotton pads serve primarily as passive barriers. They often fail to address the complex physiological requirements of healing, especially in chronic or infected wounds [7,8]. Modern wound dressings such as semi-permeable films and foams, hydrocolloids, and hydrogels [9] include interactive and bioactive materials designed to actively participate in the healing process [10]. These advanced formulations aim to control infection, modulate inflammation, promote cell migration, and deliver therapeutic agents directly to the wound site [11,12,13].

Despite their promising properties and biological effects, evaluating the optimal hydrogel formulation for wound healing is challenging due to the complexity of both the hydrogels themselves and the diverse types of wounds. Hydrogels need to perform multiple functions—such as aiding debridement, maintaining moisture, promoting cell proliferation, enhancing drug delivery, and stimulating epithelialization—throughout different stages of healing. Additionally, the wide variety of wound types—acute, chronic, infected, burn, ischemic, superficial, or deep—further complicates the selection of an appropriate testing model.

Numerous models are used to assess wound healing, ranging from in vitro systems like 2D skin cultures to more complex 3D reconstructed skin models and ex vivo human or porcine skin. Common in vivo models include surgical wounds with or without induced diabetes mellitus, burn wounds of varying depth, and, less commonly, ischemic or radiation-induced wounds on different animal species, predominantly rats and mice.

The sheer variety of models, each with advantages and disadvantages linked to complexity, costs, technical requirements, standardization issues, ethical concerns, particularly for animal and human studies, and the difficulties of clinical translation of the obtained data make it difficult to select and standardize a testing method that allows for consistent comparisons across studies.

In view of these facts, the manuscript aims to summarize current hydrogel types used in wound healing and to present an overview of the biological efficacy of various hydrogels evaluated through in vitro, ex vivo, in vivo, and clinical models. It also highlights the advantages and limitations of each approach, aiming to support more informed and effective model selection for testing hydrogel-based wound healing therapies.

## 2. Relevant Sections and Discussion

### 2.1. Hydrogels

Among the various types of wound dressings, hydrogels stand out due to their superior moisture-retention capacity, biodegradability, biocompatibility, and ability to facilitate natural debridement processes. Hydrogels are three-dimensional, hydrophilic, porous polymeric networks that exhibit high water retention capacity. They received significant attention in topical wound therapy due to their ability to mimic the native extracellular matrix (ECM), maintain a moist wound environment, and deliver bioactive agents in a controlled manner. Their structure, formed through either physical (reversible ionic or hydrogen bonds) or chemical (covalent) crosslinking, can be engineered to respond to various stimuli such as pH [14,15], temperature [16,17], light [18], chemical triggers [19], or enzymatic activity [20,21], which allows dynamic interaction with the wound microenvironment [22,23,24]. They can absorb exudate, promote oxygen diffusion, soften necrotic tissue, and provide a soothing, non-adhesive interface that enhances patient comfort and compliance. Hydrogels can replicate both the physical structure and biological functions of the ECM, supporting cell adhesion, signaling, and matrix deposition, all critical for tissue regeneration [25,26]. Hydrogels, particularly those based on biopolymers such as hyaluronic acid [27,28], collagen [29,30], alginate [31,32], or synthetic polymers like polyvinyl alcohol (PVA) [33], have emerged as promising tools in skin regeneration due to their reduced toxicity and manufacturing variability. They can act as dermal substitutes that prevent scarring by mimicking the mechanical properties of healthy skin. Also, they develop an optimal microclimate between the wound bed and the dressing [34]. Therefore, hydrogels play a fundamental role in advanced wound care strategies, offering both functional adaptability and therapeutic efficacy across a wide range of wound types.

### 2.2. Hydrogel Classification in the Context of Topical Wound Therapy

Hydrogels can be systematically classified according to several key parameters: origin or source (natural, synthetic, or semi-synthetic), crosslinking mechanism (physical or chemical), responsiveness to external stimuli (such as pH, temperature, or enzymatic activity in the case of stimuli-sensitive hydrogels), and therapeutic agent delivered. This classification approach facilitates a comprehensive understanding of the functional properties of hydrogels and supports the rational selection of suitable hydrogel systems for specific wound types and experimental biological models.

#### 2.2.1. Origin of Polymeric Scaffold

Based on the origin of their polymeric scaffold, hydrogels can be classified into natural, synthetic, and semi-synthetic [35,36].

Natural hydrogels are derived from biopolymers obtained from plant, animal, or microbial sources. Biopolymers commonly used for hydrogel fabrication include polysaccharides such as cellulose [37,38], alginate [39,40,41], chitosan [42,43,44], and hyaluronic acid [45,46,47], as well as proteins like collagen [48,49,50], gelatin [51,52,53], and fibrin [54].

Due to their structural similarity to native soft tissues and their bioactivity, natural hydrogels can support cellular adhesion, proliferation, and differentiation. Moreover, they exhibit excellent biocompatibility, biodegradability, and low toxicity, making them suitable for biomedical applications such as wound dressings, drug delivery systems, and tissue engineering scaffolds [55]. However, due to their heterogeneous molecular structures and limited molecular tunability, natural polymer-based hydrogels typically exhibit weaker and less stable cross-linked networks. This results in lower tensile strength, elasticity, and overall mechanical performance [56,57].

Synthetic hydrogels are three-dimensional polymer networks formed through the covalent or ionic crosslinking of hydrophilic homopolymers or copolymers. They offer precise control over molecular architecture due to a well-defined structure, which can be adjusted to enhance their properties. This allows tailored mechanical, chemical, and degradation properties for specific biomedical or industrial requirements [58]. Commonly used synthetic polymers include polyethylene glycol (PEG) [59,60], polyvinyl alcohol (PVA) [61,62], polyacrylamide (PAAm) [63,64], poly(hydroxyethyl methacrylate) (PHEMA) [65,66], and polylactic-co-glycolic acid (PLGA) [67,68].

Semi-synthetic hydrogels are engineered by combining natural and synthetic polymers. The aim is to integrate the biocompatibility and biological activity of natural materials with the mechanical strength, tunability, and structural stability of synthetic polymers. This leads to advanced biomaterials with enhanced mechanical performance, controlled degradation profiles, and superior biological functionality for applications in regenerative medicine, drug delivery, and tissue engineering [69,70].

#### 2.2.2. Cross-Linking Mechanism

Natural and synthetic polymers contain various hydrophilic functional groups, such as –COOH, –OH, –NH_2_, –CONH, –CONH_2_, and –SO_3_H, that facilitate the formation of three-dimensional hydrogel networks through either physical interactions or chemical crosslinking. Based on these crosslinking mechanisms, hydrogels are broadly categorized into two types: chemically and physically crosslinked hydrogels. Chemically crosslinked hydrogels are characterized by stable covalent bonds between polymer chains. Physically crosslinked hydrogels are formed through reversible interactions such as hydrogen bonding, ionic interactions, hydrophobic associations, and other non-covalent intermolecular forces [71].

Chemically crosslinked hydrogels exhibit robust three-dimensional networks with enhanced mechanical strength, elasticity, structural integrity, and prolonged degradation profiles. A variety of strategies can be employed to achieve covalent crosslinking. Common approaches include reactions between complementary functional groups (e.g., –COOH, –OH, –NH_2_), such as amide bond formation via carbodiimide chemistry [72,73], isocyanate-based addition reactions [74,75], and Schiff base reactions involving aldehydes and amines [76,77,78]. Typical crosslinkers used include glutaraldehyde [79,80], epichlorohydrin [81,82], and divinyl sulfone [83,84]. Free radical polymerization is another widely used method, where monomers bearing unsaturated double bonds undergo rapid polymerization initiated by thermal, UV, or redox initiators, allowing fast network formation under mild conditions [85,86,87]. Condensation reactions, such as those forming polyesters or polyamides, are another approach to hydrogel formation, particularly when using carboxylic acid and amine or hydroxyl groups [88,89]. Additionally, enzymatic crosslinking—such as transglutaminase-catalyzed amide bond formation—offers a biofriendly alternative, suitable for in situ applications [90,91].

Physically crosslinked hydrogels are formed through inter- and/or intramolecular non-covalent interactions between polymer chains, such as hydrogen bonding, ionic interactions, hydrophobic associations, van der Waals forces, crystallization, and protein-based interactions [92,93]. These reversible interactions allow the formation of three-dimensional networks without the need for chemical crosslinkers, making them especially attractive for biomedical applications due to their biocompatibility, injectability, and absence of toxic residues. For example, hydrogen bonds form between complementary functional groups like hydroxyl, carboxyl, and amino moieties; ionic interactions occur between oppositely charged polymers (e.g., alginate with Ca^2+^); and hydrophobic interactions drive self-assembly in amphiphilic block copolymers [92,94,95]. Additionally, crystallization (e.g., freeze–thaw treatment of PVA) [96,97] and protein–protein interactions (e.g., in gelatin or silk fibroin) [98,99] can establish physically crosslinked networks. While these hydrogels offer stimuli-responsiveness and self-healing capabilities, they often exhibit lower mechanical strength and limited stability compared to chemically crosslinked hydrogels.

#### 2.2.3. Stimuli-Responsive Hydrogels

Stimuli-responsive hydrogels can be systematically classified based on the specific external stimuli to which they respond. There is a broad spectrum of physical, chemical, and biological triggers: pH, temperature, light, electric and magnetic fields, and biological signals.

pH-responsive hydrogels, the most common type of stimuli-sensitive hydrogels, swell or shrink in response to environmental pH changes due to ionizable groups like carboxyl or amine moieties, making them particularly valuable in wound healing by enabling targeted drug release and responsive behavior in the acidic microenvironment of infected or inflamed tissues [100].

Temperature-responsive hydrogels, which undergo sol–gel transitions near body temperature, are important in wound healing, as they enable injectable, in situ forming systems that adapt to the wound site. This provides sustained drug release, improves local drug concentration, and minimizes the systemic side effects associated with intravenous or oral administration [101,102,103]. Photo-responsive hydrogels contain light-sensitive groups like o-nitrobenzyl [104], coumarin [105,106], and azobenzene [107,108] that trigger structural changes under specific wavelengths through photoisomerization, photocleavage, or photodimerization [109], enabling precise, light-controlled drug release and dynamic modulation of the wound environment, promoting targeted therapy [110]. Electro- and magneto-responsive hydrogels [111,112] react to electrical or magnetic fields, enabling controlled deformation or drug release. Glucose- and enzyme-responsive hydrogels [113,114] respond to biochemical triggers, making them ideal for targeted therapies.

#### 2.2.4. Type of Therapeutic Agent Delivered

Hydrogels have attracted significant interest as versatile carriers in targeted therapeutic delivery, bioadhesive systems, and controlled release platforms. Due to their inherent biocompatibility, moisture retention, and structural similarity to the extracellular matrix, hydrogels, in particular polysaccharide-based, are highly effective for wound healing applications, even in carrier-free formulations. They support tissue regeneration and maintain an optimal healing environment with no need for additional therapeutic agents [115,116]. Hydrogel-based wound dressings have been extensively explored for the delivery of therapeutics such as antimicrobials, growth factors, nucleic acids, metal nanoparticles, and exosomes. These systems enhance drug stability in the intracellular environment and prolong therapeutic activity at the wound site, even for periods exceeding 10 days.

Growth factors and peptides promote cell proliferation and angiogenesis but suffer from rapid degradation, while hydrogels protect and sustain their release [117,118,119]. Nucleic acid therapeutics undergo enzymatic breakdown and exhibit poor uptake, yet hydrogels improve their stability and transfection efficiency [120,121]. Exosomes boost immune modulation and regeneration; hydrogel incorporation extends their retention and therapeutic effect [122,123]. Antimicrobials are essential for infection control but often suffer from low bioavailability, burst release, and potential toxicity. Incorporation into hydrogels enhances their bioavailability through localized, sustained release. It minimizes systemic exposure and reduces side effects and resistance [124]. Metallic and non-metallic nanoparticles offer antimicrobial, antioxidant, and healing benefits, while hydrogels ensure their even distribution and prolonged activity, addressing complex wound care challenges [125,126].

Based on these facts, the use of hydrogels in wound healing care, regenerative medicine, or drug delivery presents clear advantages. However, their biocompatibility, biological activity, or efficacy strongly depends on the hydrogel interactions with the biological tissues. Choosing the right model is essential for the testing of the hydrogel properties, since their use might be beneficial in a certain wound type or a phase of the healing process, while it might be detrimental in another step.

For instance, a hydrogel that exerts anti-fibrotic properties, such as alginate, has a negative impact in the proliferation step but can be beneficial in the maturation phase or in chronic wounds when fibroblast overactivation leads to epithelialization inhibition [127]. Alginate is widely used in wound care in forms such as hydrogels, films, nanofibers, microspheres, and topical formulations [128,129,130,131]. It is a biocompatible, linear anionic polysaccharide derived from brown algae (e.g., Sargassum) and some bacteria. Alginate hydrogels can be formed via various crosslinking methods—ionic, covalent, thermal, or cell-mediated—allowing customization of their physical and chemical properties for biomedical applications [132].

In the inflammatory phase of wound healing, a non-absorbent hydrogel, like naturally derived polymers such as collagen or alginate-based formulations, is less useful due to abundant exudate but is beneficial in the proliferation and migration phase, when it provides an ECM-resembling scaffold that diminishes healing time and improves scar formation. Alginate films could also be unsuitable for heavily exuding wounds due to poor thickness [129]. Aloe vera-enhanced alginate films improved mechanical strength and thermal stability without altering chemical properties [133]. Alginate microspheres can be used as delivery systems for drugs, growth factors, and cells [134,135,136], or to enhance the absorbance properties of the polymer while still maintaining moisture in the wound bed [127,131].

From a translational perspective, natural polymers such as alginate, chitosan, hyaluronic acid, collagen, and gelatin are attractive due to their biocompatibility, biodegradability, and intrinsic bioactivity. However, they also exhibit disadvantages such as poor mechanical strength and the risk of immunogenicity, which limit direct clinical translation unless combined with reinforcing networks or antibacterial agents. In contrast, synthetic systems such as polyvinyl alcohol (PVA) hydrogels provide robust mechanical stability, moisture retention, and oxygen permeability. These properties make them suitable for protecting fragile wounds and promoting re-epithelialization, despite their lack of inherent antibacterial activity, which requires functional modification. Several reviews have comprehensively examined the advantages and limitations of both natural and synthetic polymer-based hydrogels for wound healing applications as well as clinically available hydrogel wound dressings [137,138,139].

Hybrid hydrogels bridge these gaps by integrating bioactive natural polymers with mechanically resilient synthetic backbones or responsive moieties, enabling multifunctional dressings that can release antimicrobials, respond dynamically to wound microenvironmental cues, and support cellular proliferation in vivo. Such adaptability, coupled with a high potential for scalable and low-cost manufacturing, positions hybrid and engineered synthetic hydrogels as the most clinically translatable platforms for wound care applications [140].

Hybrid hydrogels containing polyvinyl alcohol (PVA), with chitosan with or without collagen, can be used in all healing phases due to their dual effects of absorbing and moisture preservation, which regulates the humidity of the wound bioenvironment [141]. Nanocomposite sponges using sodium alginate, graphene oxide, and PVA showed high porosity, good mechanical properties, stimulated cell proliferation in vitro, and enhanced wound healing in vivo [142]. The use of hybrid hydrogels in wound healing is still limited by mechanical properties, biocompatibility, and the formation of degradation byproducts, such as acids. For instance, polylactic acid (PLA)-containing gels decrease tissue pH by freeing lactic acid, which is detrimental for cell survival and proliferation [143].

Cellulose also proved beneficial due to its mechanical strength and absorptive ability. Carboxymethyl cellulose and electrospun cellulose extracts with polylactide and polydioxanone nanofibers increased skin regeneration and aided angiogenesis, reviewed in [143].

Polyurethane-based wound dressings, incorporating natural polymers (e.g., collagen, chitosan, hyaluronic acid), synthetic polymers (e.g., PEG, PVA, polyacrylamide), and bioactive agents (e.g., LL37 peptide, platelet lysate, exosomes), have been reviewed for their physicochemical and biological modifications, applications in wound repair and regeneration, and potential for the development of advanced functional dressings [144]. Bilayer dressings combining a dense polyurethane membrane with a polycaprolactone/gelatin scaffold control wound moisture, provide antibacterial activity, and promote cellular adhesion and proliferation, supporting multiple stages of wound healing [145].

Hydrocolloid dressings were prepared from sodium carboxymethyl cellulose or sodium alginate, styrene–isoprene–styrene, and either silk fibroin nanoparticles or petroleum hydrocarbon resin, optionally loaded with bioactive agents. These hybrid hydrocolloids exhibit improved mechanical properties, water uptake, and swelling. They promote dermal regeneration and epithelialization, enhance wound structural integrity, reduce wound size, and increase collagen fiber density [140].

Another issue in wound healing is the presence of bacteria, leading to inflammation and cell death, with delayed or impaired healing eventually leading to the development of chronic wounds or defective scars. Composite hydrogel dressings with carboxymethyl chitosan, gelatin microspheres, and tetracycline hydrochloride showed sustained drug release and effective inhibition of *E. coli* and *S. aureus* [146]. Innovative blends, such as silk fibroin/sodium alginate membranes loaded with strontium, promoted angiogenesis and exerted antibacterial effects by disrupting bacterial functions [147].

Nanoparticle incorporation further enhanced antibacterial activity [148]. Mercy et al. synthesized combined hydrogels containing alginate/gelatin hydrogels with nanosilver with or without plant extracts such as aloe vera, curcumin, plantain, and Calendula. The hydrogels showed low cytotoxicity, strong antibacterial activity against *E. coli* and *S. aureus*, and enhanced wound closure in vitro on fibroblastic cells (V79). Adding the plant extracts increased efficacy [149].

Microencapsulated plantain extract in alginate promoted antioxidant and anti-inflammatory effects, boosted collagen synthesis in dermal fibroblasts (BJ), and accelerated in vitro wound closure [131]. Alginate microspheres containing white lily extract showed increased in vitro wound healing effects by antioxidant and anti-inflammatory activity and the modulation of matrix metalloproteases, while boosting collagen synthesis on a co-culture model of senescent dermal fibroblasts (BJ) and endothelial cells (HUVEC) exposed to LPS. The model mimicked a chronic, infected wound [127]. Sodium alginate beads, containing resveratrol with or without graphene nanoplatelets, were synthesized and tested for physicochemical properties, resveratrol release, and biocompatibility in human cell culture. The gel beads showed good biocompatibility against dermal fibroblasts (BJ) and endothelial cells (HUVEC) and provided an antioxidant effect by long, pH-dependent resveratrol release [146]. Moringa oleifera leaf (MOL) extract, rich in antimicrobial and anti-inflammatory compounds, was incorporated into polyvinyl alcohol (PVA) and graphene oxide (GO) hydrogels. The PVA/MOL/GO hydrogel exhibited strong antibacterial activity against *S. aureus* and *E. coli* and cytocompatibility in 3T3L1 cells and shortened in vitro wound healing, as confirmed by scratch assay [150]. SA-based hydrogels—sulfonated, quaternized, and chlorogenic acid (CGA)-modified—were coated onto Eucommia ulmoides rubber (EUR) films. Biocompatibility was confirmed using L929 fibroblasts. The dressings showed good mechanical strength, water vapor permeability, and controlled drug release. Antibacterial tests against *E. coli* and *S. aureus* and an in vivo mouse full-thickness wound model revealed accelerated healing, confirmed by histological and immunohistochemical analyses [151].

There is a growing need for suitable biological models that are specifically designed for the testing of hydrogel properties in the bioenvironment, which would allow the fine-tuning of their physico-chemical structure to the intended use. These models have to mimic the clinical steps of wound healing, depending on the wound types and the behavior of different cell types from the wound area.

In view of the clinical use of hydrogel formulations, their biocompatibility and biological activities have to be thoroughly tested on human cell-derived models, closely mimicking the wound structure. The employed models involve in vitro 2D skin cell cultures and possibly more complex human models, such as 3D reconstructed human epithelium or full-thickness skin models, ex vivo human or porcine explants, or standardized in vivo models created on lab animals, predominantly mice and rats.

### 2.3. Biological Models Employed in the Evaluation of the Wound Healing Properties of Hydrogels

#### 2.3.1. In Vitro Models

In vitro wound healing models (Table 1 and Table 2) play a vital role in improving our understanding of tissue repair by providing a simplified, controlled environment, enabling systematic testing of therapeutic interventions under reproducible conditions.

A comparative presentation of the advantages/disadvantages, limitations, and research applications of the in vitro models is presented in Table 1 (2D models) and Table 2 (3D models).

##### Monolayer Cultures

**Figure 1:**
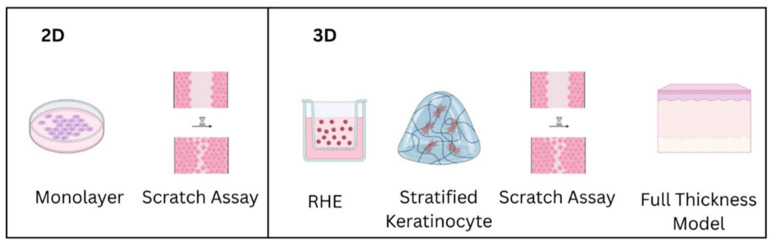
The most relevant in vitro wound healing models. Two-dimensional models (**left** panel), represented by monolayer cultures of fibroblasts/keratinocytes with or without adding other cell types such as endothelial cells (for vasculature) and immune cells (for inflammation studies), and the scratch assay represent useful tools for assessment of wound healing, with multiple advantages, such as high reproducibility, simplicity, and cost-effectiveness, at the expense of limited complexity. Three-dimensional models (**right** panel), represented by stratified keratinocytes, reconstructed human epithelium (RHE), and full-thickness skin models (FTM), mimic the complexity of skin epidermal layers with or without the dermal layer and add other skin cells, such as endothelial, immune, or neuronal cells, to increase the physiological relevance at the expense of increased costs and enhanced production and assessment difficulties.

Fibroblast and/or keratinocyte monolayer models are simpler models, easier to manipulate, and provide cost-effective benefits. Cells are cultured cells on flat and rigid surfaces generally made of transparent glass or plastic, so microscopic cellular observation is possible while maintaining cellular attachment [152]. One of its shortcomings is that the monolayer lacks three-dimensional structure and therefore complex cell–cell interactions and ECM dynamics in actual tissues, restricting its physiological interactions and diminishing the possibility for direct clinical transfer. However, the 2D in vitro models (Figure 1) are frequently used for basic research into cellular mechanisms such as preliminary drug screening and toxicity assessments [153], cell migration and basic proliferation studies, and cell signaling pathways in wound repair analysis [152].

An interesting approach to wound healing modeling was the development of a human-relevant in vitro scab model composed of HaCaT keratinocytes and fresh human blood, maintained for over two weeks. The model expressed all TGF-β isoforms and ECM-related target genes, reflecting early wound healing activity. TGF-β1 stimulation promoted matrix remodeling and Smad7 expression, while pathway inhibition led to scab condensation and degranulation. This model provides a viable platform to study early wound healing events involving blood–skin cell interactions [161].

##### Scratch Wound Assays

The scratch assay is a 2D in vitro technique derived from the monolayer model, wherein a uniform “wound” is introduced by mechanically disrupting a confluent layer of cells, typically epidermal fibroblasts or keratinocytes. Cell migration into the wound area is tracked over time to assess wound closure. Some of the most common uses of this method are in wound healing and cancer assays.

Another wound creation technique comes from the use of inserts, including silicone or bioprinted, to avoid the mechanical trauma inflicted on neighboring cells [154]. The scratch wound assay is valued for its reproducibility and simplicity and can be quantified by imaging software, including available open-source analytical platforms [155]. However, it lacks the structural and functional complexity of the in vivo tissue, offering limited insight into ECM dynamics, dermal-epidermal interactions, and immune responses [152]. The method was also developed for 3D models. Initially a 3D fibrin clot scaffold was used for cell migration; then collagen or other matrices and fibroblast spheroids were employed to test fibroblast and neutrophil migration in the presence of inhibitors or activators [156]. A more precise scratch assay method was developed based on photosensitized culture substrates. Photosensitizers were engulfed into a polystyrene matrix, which was coated in a thin layer on the bottom of the cell culture dish. After the confluent culture was grown on the dish surface, the photosensitizer was activated by irradiation, which produced superoxide anion that killed the cells precisely in the exposed area, leaving a precise wound. The method can be applied to monolayers, 3D cultures, or even microfluidics to generate the wounds [157].

##### Reconstructed Human Epidermis (RHE), 3D Models

Researchers have developed more advanced models, such as the 3D reconstructed human epidermis (RHE) or full dermal-epidermal reconstructs (FDE), to overcome the 2D tissue architecture limitations [152].

RHE models consist of stratified keratinocytes cultured on a porous membrane at the air-liquid interface, frequently supported by a collagen matrix to mimic the basal membrane. Full dermal-epidermal reconstructions also include the reconstructed dermis by using human fibroblasts for the dermal part, on which the keratinocytes are further stratified to make the upper epidermis. These models allow for a multilayered epithelium with structural and functional similarities to native human skin, including barrier function and ECM deposition [152]. The air-liquid interface promotes keratinocyte differentiation and stratification, resulting in a tissue architecture more suitable to represent in vivo conditions. As a general use, these models allow research focused on tissue engineering and regenerative medicine, drug efficacy testing, or studies involving complex cell behaviors within a physiologically relevant environment. RHE and FDE models have high immediacy in studies focused on epidermal development, barrier stability, and preclinical testing of topical agents [162]. However, these benefits come with increased complexity, higher expenses, and the need for specialized equipment and expertise (Table 2 and Table 3). Additionally, their viability is limited to shorter timeframes, and analytical procedures may be more challenging due to their 3D nature [163].

An in vitro thermal wound infection model was developed using human skin equivalents (HSE) and biofilm-forming MRSA to evaluate antimicrobial agents. Thermal injury, induced by a liquid nitrogen-cooled device, caused keratinocyte death and epidermal detachment, triggering inflammation and antimicrobial responses. MRSA exposure led to rapid colonization and upregulation of TLR2, IL-6, IL-8, and defensins. Topical mupirocin reduced MRSA levels by over 99.9% within 24 h. This model effectively mimics early wound infection and is suitable for testing topical antimicrobials [164].

Hydrogels, particularly collagen, are useful tools for the design of the dermal matrix or as a bioink to support the development of the 3D RHE or full-thickness skin models. A full-thickness human skin model (FTM) was developed using chitosan and collagen hydrogel (CC-FTM). The CC-FTMs supported epidermal differentiation, with reduced keratinocyte activation resembling normal human skin. It also induced an improved lipid matrix structure, ceramide profile, and lower transepidermal water loss, which indicated enhanced barrier function. These features make CC-FTMs a more physiologically relevant model for studying epidermal barrier properties [164]. A multicellular wound-on-chip model was developed to mimic the paracrine signaling of early inflammation during wound healing. Using collagen or Matrigel coatings, dermal fibroblasts and HUVECs were cultured in a tri-channel microfluidic system. Inflammation was induced by TNF-α or macrophage triculture, both significantly increasing IL-1β, IL-6, and IL-8 levels, which were reduced by dexamethasone. M2 macrophages enhanced cytokine production and vascular structure formation. This model effectively replicated early wound inflammation [158].

An overview of the wound healing processes in 2D and 3D in vitro models is provided in Table 3.

#### 2.3.2. Ex Vivo Models—Human or Porcine Skin Explants

Ex vivo skin models involve culturing excised human or animal skin to replicate the complex structure and microenvironment of native tissue. They are widely used to study wound healing mechanisms and inflammation, and to assess the biological effects of therapeutic interventions. A key advantage is the preservation of the original skin architecture and cell-to-cell communication. It presents the stratified epithelial organization while also maintaining the epidermis, dermis, and basement membrane layers. Ex vivo models also show communication between fibroblasts, keratinocytes, and extracellular matrix proteins. This allows for more physiologically relevant observations compared to in vitro cultures. However, these models lack vascularization, innervation, and immune cell migration, limiting their ability to accurately replicate the dynamic immune responses seen in vivo, such as the recruitment of circulating leukocytes, cytokine signaling cascades, and the resolution of inflammation. Furthermore, variability in tissue sources and preparation methods may limit reproducibility and long-term applications [159].

In a human skin explant model mimicking superficial wounds via tape-stripping, the topical application of octenidine dihydrochloride-based hydrogel promoted wound healing by lowering inflammatory and proteolytic activity, shown by reduced cytokine and matrix metalloproteinase expression [160]. Similarly, an ex vivo porcine skin model treated with a thyme-oil-loaded PLGA (polylactic-co-glycolic acid) nanoparticle hydrogel increased healing by antioxidant mechanisms [165]. A more complex human skin model simulating deep burn wounds under tension revealed that p407/p188 poloxamer hydrogel slowed epidermal regeneration [166]. In another study using partial-thickness human skin wounds, a polydopamine-based hydrogel with in situ generated silver nanoparticles promoted epidermal regeneration, improved biocompatibility, reduced inflammatory markers, and demonstrated broad-spectrum antimicrobial activity [167].

In an ex vivo porcine skin model treated with adamantane-based collagen mimetic peptide (CMP) hydrogel, the hydrogel showed satisfactory permeation for the peptide, leading to its localization in the epidermis and dermis [168]. Complex alginate hydrogels loaded with curcumin-synthesized cerium oxide nanoparticles (Cur-CeO) showed good biocompatibility to cultivate L929 fibroblasts and antioxidant properties ex vivo. The hydrogel-nanocomposites accelerated circular deep wound repair by reducing oxidative stress and inflammation and increasing keratinocyte and fibroblast migration onto the wound bed to promote faster tissue recovery in rats [169].

In vivo models involve various animal species such as mice, rats, rabbits, and guinea pigs. Common in vivo models include surgical wounds (incisional or excisional), diabetic non-healing wounds (induced via chemical methods), burn wounds of varying depth, and, less commonly, ischemic or radiation-induced wounds.

#### 2.3.3. In Vivo Wound Healing Models

**Figure 2:**
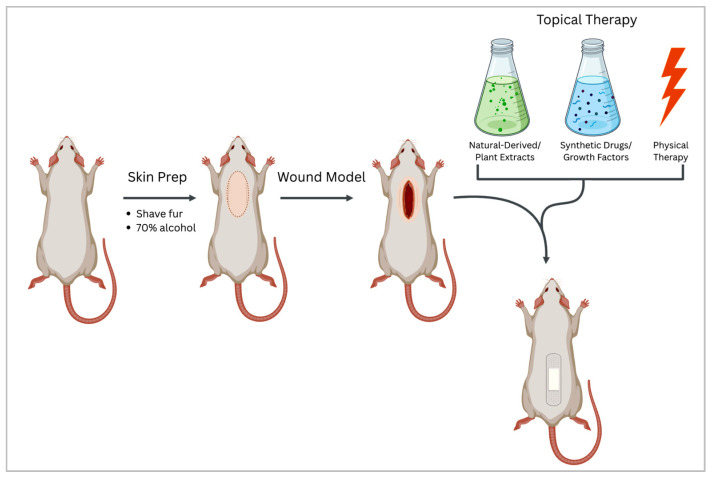
Schematic diagram of the design of surgical wound models in vivo for the study of healing efficacy of different types of chemical and physical treatment options. Created with www.BioRender.com, accessed on the 9 July 2025.

Several models can be employed to create a standardized wound in vivo (Figure 2, Table 4, Table 5 and Table 6). Surgical wounds can be performed as full-thickness excisional wounds or incisional wounds. One of the most used is the full-thickness excisional wound, when a circular or square wound is excised down to the panniculus carnosus (in rodents) or subcutaneous tissue. It is easy to perform, standardize, and monitor throughout the healing phases. Incisional wounds consist of linear cuts, sometimes sutured, to mimic surgical incisions and test healing strength and scar formation. The wound size is usually standardized, e.g., 6–10 mm diameter for mice [170,171].

Another type of wound is the burn wound, which is a thermal injury induced to study scarring, delayed healing, or infection. Special types of models can be created using ischemia or diabetic wounds when the animals, usually mice or rats, have a previously induced diabetes mellitus condition (e.g., streptozocin STZ -induced diabetic mice). These models mimic chronic or non-healing wounds [171].

##### Surgical Wounds

The in vivo surgical wound model is a widely used technique to study wound healing processes under controlled biological conditions. Surgical wounds can be developed in animal subjects (mice, rats, guinea pigs, rabbits, pigs, or larger animals) and simulate different types of wounds to assess tissue regeneration, epithelialization, healing mechanisms, therapeutic interventions, scarring, and inflammation. Lab animals typically used for these kinds of models are mice and rats due to the low costs, easy handling in the lab, and well-characterized phenotype, including the immune response (Table 4, Figure 2).

The efficacy of different gel formulations containing naturally derived antioxidants [173,175,176,177,183], sodium thiosulfate, a ferroptosis inhibitor [174], sildenafil cream [179], or nanoliposomes containing spirulina-derived peptides [193] on mouse or rat models is presented in Table 4.

**Figure 3:**
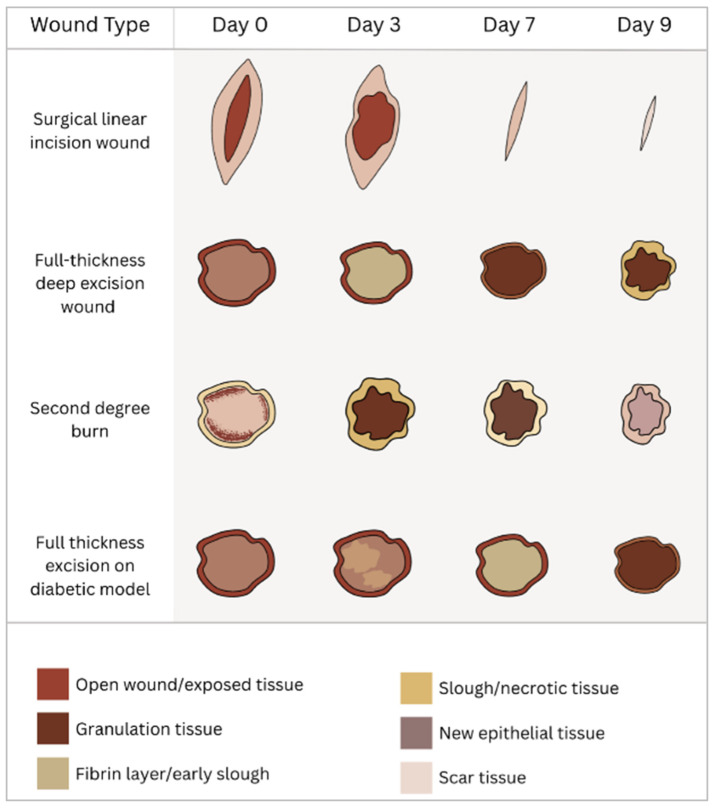
Comparative wound healing timeline for different types of in vivo models. All wound models show healing and/or scarring on day 9, except for the diabetic wound. Created with www.BioRender.com, accessed on the 9 July 2025.

Diabetic wounds heal poorly due to peripheric angiopathy and neuropathy-induced changes and chronic inflammation [197,198], (Figure 3). Some studies showed emerging promising therapies that address these specific pathological barriers and promote functional tissue repair by using paeoniflorin-loaded hyaluronic acid (HA-PF) hydrogel [187] or N-acetyl cysteine [186].

In nonhealing surgical wounds, an efficient approach was the local therapy with extracellular vesicles, cellular fractions, and ECM embedded in gel formulations. The advantages are multiple: low immunogenicity, since they contain no intact cells; well-preserved vesicles, which allow transport of the active compounds through the wound bed; and direct cell stimulation by growth factors [178,188,189,192].

Developing an antibacterial hydrogel for the therapy of surgical wounds, particularly efficient against Staphylococcus aureus, a common pathogen found in the wound bed of humans and animals, was attempted by incorporation of ZnO nanoparticles in a double-layer alginate dressing [199], silver nanoparticle-coated alginate nanofibers [200], or lavender oil-infused nanofiber matrices [201].

Another issue that needs to be solved when using hydrogels for local therapy on in vivo animals or humans is their mechanical properties, particularly stiffness, adhesion, and moisture retention characteristics. These properties are required for the initial application of the hydrogel and afterwards for the persistence of the gel on the wound. An interesting approach was the development of an injectable, self-healing hydrogel composite (OGN-7) using oxidized alginate, gelatin, and 5 mg/mL PCL:gelatin nanofibers (7:3 ratio). Dual Schiff-base crosslinking and nanofiber integration enabled in situ gelation without additional crosslinkers. OGN-7 demonstrated enhanced stiffness, faster gelation, excellent remendability, and biocompatibility. It fully fused within 1 h, maintained mechanical integrity under stress, and outperformed standard alginate dressings in a full-thickness wound model. Histological analysis confirmed improved re-epithelialization and collagen deposition [202].

Another approach is the patch application directly on the wound bed because of the adhesive, hydration, drug delivery, and antimicrobial properties of these complex formulations, such as chitosan with doxycycline, zinc, and selenium nanoparticle patches [184], chitosan membranes [185], or silk wraps with epidermal growth factor or silver sulfadiazine [194] (Table 4).

Stimulation of wound bed cell proliferation can be performed by local physical therapies such as laser [190], photodynamic therapy (PDT) [196], electrical current [195] (Figure 2).

Moreover, mutations can be induced in mice to generate adequate models for the study of healing mechanisms. Systemic interleukin-10 administration improved wound healing, reduced inflammation, and enhanced scar quality in KO mice with double deletion of IL-10 and IL-4 genes, proving the important role of IL-10, an anti-inflammatory cytokine, in the normal healing process [191].

The use of rabbits for the generation of surgical wounds is beneficial since it allows a larger wound area, which is more useful for the efficacy and biocompatibility of dressings or biomaterials. In a recent study, a nanoemulsion hydrogel with Hypericum perforatum upregulated growth factors, increased vessel growth, and showed antimicrobial characteristics in rabbits [172]. A similar antibacterial effect was obtained in rat and rabbit models; using Aloe ferox Miller and Aloe arborescens Miller promoted healing, reduced inflammation, and displayed selective antimicrobial activity [181].

##### Burn Wounds

Burn wounds represent a model mainly used to study the scarring in different conditions in a chronic or nonhealing/infected wound (Figure 3). However, a significant problem with the burn wound model is the standardization, which makes comparison between different therapies difficult. Although each study has its own control group, the burn depth and wound surface are important features that influence the healing process. For instance, a full-thickness burn heals slower and with scarring, due to the lack of hair follicle stem cells, when compared to a second-degree burn. Also, some of the studies do not offer complete data regarding the wound dimensions, such as depth, diameter, closure timeline, and scab formation. When compared to burn wounds, surgical wounds can be standardized, and the follow-up can be performed easier, leading to more comparable results. Despite these issues, the advantages of the burn wounds when following a strict protocol are significant: they can be easily created, the method is quick and reproducible, it is less stressful, and the survival of the animals is very good when compared to a diabetic model with a surgical wound that mimics a similar chronic nonhealing wound.

There are several studies that used this model to evaluate the results of local hydrogel therapies such as chitosan gel with epidermal growth factor (EGF) [203], zinc alginate hydrogel [204], verapamil-loaded hydrogel with EGF and verapamil nanofibers alone [205], and Zein/pectin/Vitamin C scaffold crosslinked to mimic hydrogel [206]. The results are presented in Table 5. All formulations showed improved healing time and led to better scar formation when compared to controls. Adding the epidermal growth factor was beneficial when combined with chitosan but not with verapamil, while the presence of the vitamin C enhanced collagen 1 expression. An interesting approach was the use of Clostridium Perfringens Wound Healing Substance to increase collagen production and capillary formation [207]. The natural antioxidants embedded in ointment [208] or administered as a spray [209] also seemed to improve healing, particularly by anti-inflammatory effects (Table 5).

**Table 5:** In vivo thermal burn wound models used for experimental research.

No.	Localization/Species	Treatment	Main Results
1	Dorsal area—rats	Zinc alginate hydrogel	Faster wound healing, increased angiogenesis, and fibroblast migration [204]
2	Dorsal area—rats	Gel containing verapamil HCl-loaded nanofibers composed with alginate	Controlled release of verapamil leading to smooth, scar-free wound closure [205]
3	Dorsal area—mice	Zein/pectin/vitamin C scaffolds crosslinked to mimic hydrogel	Antioxidant effects, faster re-epithelialization, anti-inflammatory activity [206]
4	Second-degree burn on dorsal area—rats	Chitosan gel with epidermal growth factor	Increased cell proliferation and epithelialization, improved scar tissue formation [203]
5	Dorsal area—rats	Cream containing Clostridium Perfringens-derived wound-healing substance	Enhanced skin collagen formation and increased capillary formation [207]
6	Dorsal area—BALB/c mice	Ointment containing Aloe emodin and resveratrol	Faster wound healing by increasing blood vessel growth, upregulating IL-1β and VEGF, and stimulating immune cell response [208]
7	Second-degree burn on dorsal area—rats	Spray formulation of Olea europaea and Aloe vera leaves, Cocus nucifera fruit, and Chamomilla recutita flower plant extracts	Antioxidant and anti-inflammatory effects, increased angiogenesis, faster wound healing [209]

##### Other Wounds

Research on complex wounds (ischemic, irradiated, deep, axial flap) showed improved healing by using topical application of platelet-derived [210] or tooth pulp stem cell vesicles [203], respectively, and local physical therapies such as photodynamic therapy and pulsed therapeutic ultrasound [211], the main findings are presented in Table 6.

**Table 6:** In vivo ischemic or irradiation wound models used for experimental research.

	Model/Species	Treatment	Main Results
1	Ischemic wound in rabbits	TA Platelet-derived exosome product incorporated into a surgical fibrin sealant biogel	Boosted deep tissue repair by enhancing new vessel growth, structural rebuilding, and skin appendage renewal [210]
2	Fasciocutaneous flap on rats exposed to ischemia/revascularization of the vascular pedicle	LT photodynamic therapy mediated by Photofrin and 630 nm light	Inhibited revascularization in axial flaps, showing reduced blood flow [212]
3	Ischemic wound by using the Matrigel plug model on nu/nu mice	TA apoptotic extracellular vesicles from tooth pulp stem cells	Enhanced angiogenesis by collagen 1 delivery form vesicles and stimulation of the PI3K/AKT/VEGF pathway leading to faster wound healing [205]
4	X-ray irradiation on mice	LT-pulsed therapeutic ultrasound	No beneficial effects on wound closure rate were reported [211]

Angiotensin (1–7) with systemic administration accelerated wound healing in multiple models, including rats, diabetic mice, and guinea pigs—by maintaining flap viability and improving tissue regeneration and growth, without hypertensive effects [180].

Limitations: species differences, ethical concerns

While animal models remain a cornerstone of wound healing research [213], several key limitations affect their translational relevance.

Rodents, commonly used in experimental models, differ significantly from humans in skin architecture, immune responses, and wound healing mechanisms [214]. For example, they primarily repair wounds through contraction, unlike the re-epithelialization process predominant in human skin, which limits the applicability of findings to clinical settings [215]. Also, the presence of fur increases the number of skin hair follicles, the source of the follicular stem cells, leading to quicker healing, limited inflammation, and less scarring. These properties also make it difficult to generate a standardized chronic nonhealing wound in a rodent model.

In addition, ethical concerns regarding animal use require strict regulatory oversight and justification of experimental design, in line with the 3Rs principle—Replacement, Reduction, and Refinement [216].

Model selection must also account for practical considerations such as complexity, time, cost, and the researcher’s expertise. Ethical concerns, especially with in vivo animal models and human tissue use, are significant factors as well.

Another major challenge lies in translating results from laboratory models to clinical practice. Data from in vitro studies often fail to replicate in vivo due to the intricacies of living organisms, including immune responses, hormonal regulation, and blood circulation. Furthermore, outcomes from animal studies may not accurately reflect human biology, leading to differing therapeutic effects in clinical settings compared to preclinical models.

### 2.4. Hydrogels in Clinical Practice

Hydrogels used in the treatment of different wound types present important characteristics that may help in healing [217]: adherence to the lesion but with slight removal, transparency [218]; hydration or siccative network [219]; stimulators of keratinocyte and fibroblast proliferation and migration, angiogenesis trigger [220], immune cell modulators [221]; antibacterial properties [222]; non-immunogenic structure [223]; and many other advantages if compared with classical treatments.

Nowadays, skin wounds (burns, diabetic ulcers, venous ulcers, accidents, etc.) can be treated with hydrogels applied on the lesion area as solid sheets, semi-liquid (amorphic) layers [222], foams, or sprays, including different substances that help the healing. Many polysaccharide-based hydrogels were developed with alginate, starch, cellulose, chitosan, hyaluronic acid, and carboxymethyl chitosan, but with low antibacterial activity. The usage of essential oil extracts increased the antibacterial properties of natural hydrogels, with the ginger, cumin, or eucalyptus essential oils showing the best antibacterial activities against *S. aureus* and *E. coli*, compared to other studied natural oils [224]. For antibacterial effects, metallic oxides were also introduced in some hydrogel formulas: zinc oxide, titanium dioxide, or nickel oxide [225]. The best results in wound protection were obtained with the antibiotic inclusion, such as ciprofloxacin against *S. aureus*, *E. coli*, and Klebsiella pneumoniae [226]; gentamicin against Staphylococcus saprophyticus [227]; ampicillin sodium against G+ and G− bacteria [228]; levofloxacin for the infection with *P. aeruginosa* or *S. aureus* [225]; cephalosporins against *E. coli* [225,229]; and vancomycin as the final choice for antibiotic treatment [230].

Different adjuvants of skin wound healing were introduced in hydrogels, growth factors like platelet-derived growth factor (PDGF) [231], vascular endothelial growth factor (VEGF) [232], or fibroblast growth factor (FGF) [233]. Several hydrogel formulas were developed with collagen or hyaluronic acids, molecules that induce immune reactions in human patients [234].

Nanoparticle-loaded hydrogels (metal nanoparticle hybrid hydrogels) were developed for acute (surgical, burns, etc.) and for chronic (diabetic, dermatological, etc.) wounds, patches that are enriched with silver, gold, or zinc oxide nanoparticles in hydrogel formulations that may help in tissue regeneration through their antibacterial action and drug delivery enhancement [235]. Different combinations were developed, like silver nanoparticles with epigallocatechin gallate that enhance the growth factors expression at the lesion area [236] or natural hydrogels with gold nanoparticles that can stimulate the fibroblast migration and the inflammation decrease [237].

Hybrid nanogels may adjust their characteristics (hydrophobic or hydrophilic enhancements, volume variations) to different body changes (glucose level, tissue or blood pH, temperature, biomarkers, etc.) or to environmental modifications (electrical field, magnetic field, light, etc.), features that make them efficient materials in wound treatment [58,238].

Nanoconfined hydrogels with different nanosheets are developed, with improved properties that permit the reorganization of their structure when damaged, synthetic skin that may be used in quick repairs of human skin wounds [239].

Some hybrid hydrogels are already used in patients, but there are concerns regarding their toxicity, the nanoparticles’ migration from the wound level, and their tissue accumulation (skin, liver, kidneys, etc.) [240].

#### 2.4.1. Hydrogels in Preclinical Studies

The hydrogels’ effects on wounds are investigated in many different animal experimental models. To evaluate their antibacterial activity, in vivo studies used quaternized chitosan–matrigel polyacrylamide [241], chitosan–polyvinyl alcohol [242], collagen-hyaluronic acid [243], agar-fumaric acid-silver nanoparticles [244], poly(lactic-co-glycolic acid)/polyethylene glycol/silver [245], or quaternized chitosan/curcumin/benzaldehyde terminated. The antioxidant activity of these innovative products was also investigated in animal models using gels with different compositions like PGE_2_/chitosan [246], pearl peptides/selenium-containing block-functionalized PEG/polypropylene glycol [247], or resveratrol/(collagen-laminin-based dermal matrix)/hyaluronic acid-dipalmitoylphosphatidylcholine [248].

Hydrogels able to accelerate the tissue reorganization during the healing process were developed and studied in vivo: hyaluronic acid-paeoniflorin [187], collagen-hyaluronic acid [243], feruloyl-modified peptide/glycol chitosan [249], tannic acid/PVA (polyvinyl alcohol)/PEG (polyethylene glycol)/chitosan carboxylate/hyaluronic acid [250], dopamine-substituted multidomain peptide [251], and chitosan/alginate/vitamin E [252].

#### 2.4.2. Hydrogels in Clinical Trials

The beneficial or noxious effects of hydrogels must also be studied through clinical trials, and among the experimented formulas, the following have to be mentioned: hydrogel/nano-silver-based dressing [253] or *Olea europaea* leaf extract hydrogel (EHO-85) [254] used for increasing re-epithelialization, hydrogel-urea-papain with efficacy on different lesion types [255], hydrogel enriched with sodium alginate and Vitamins A and E that showed inconclusive results [256], and *Triticum vulgare* extract-polyhexanide (Fitostimoline^®^) with good effects on inflammation but without complete healing of lesions [257]. For skin ulcers (diabetic, venous), good results were recorded when collagen with fibroblasts and keratinocytes was used as gauze applied on wounds [258]. A double-blind clinical trial conducted on 316 patients, aged above 75 years, with chronic nonhealing venous insufficiency ulcers and without diabetes mellitus or cardiovascular-associated diseases, showed significant improved responses when human-derived adipose tissue mesenchymal stem cells were used, in comparison with the control group that was treated with saline. The authors hypothesized that the immune response inhibition induced by delayed maturation of dendritic cells and natural killer (NK) cells could be responsible for the improved results of the treatment on these wounds, reviewed in [259]. A recent study, performed in patients with chronic deep wounds, compared the standard treatment with acellular dermal matrix (ADM), applied topically as a paste. The study was conducted for 12 weeks in 86 patients aged above 19 years. Patients with complicated wounds (infections, tunneling), diabetes mellitus, or superficial wounds were excluded. The results showed wound healing in 76.3% of cases in the experimental group, compared to 30.6% in the control group. These outcomes represent a promising therapy option for chronic nonhealing wounds [260].

#### 2.4.3. Approved and Commercialized Gels

Since hydrogels contain different types of polymers, the US FDA (Food and Drug Administration) and MDA (Medical Device Amendments) incorporated these innovative treatments in class I, II, and III of risk for developing diseases related to the drug compounds.

##### Synthetic Wound Dressings

For dry skin lesions, several synthetic wound dressings are recommended: 3M™ Tegaderm™ hydrogel wound filler (propylene glycol), DermaSyn or Cutimed gel (carbomer 940), Woun’Dres (carbomer, collagen), DermaGauze™ (acrylate polymer), and Simpurity™ Hydrogel (polyethylene oxide, polyvinyl alcohol, acrylate, polyurethane). The management of ulcers and burns may be performed with Suprasorb G (CMC polymer, propylene glycol) or Simpurity™ Hydrogel (polyethylene oxide, polyvinyl alcohol, acrylate, polyurethane), and of radiation skin lesions with AquaDerm™ (2-Acrylamido-2 methyl-1-propanesulfonic acid sodium, propylene glycol, poly(ethylene glycol) dimethacrylate, 2-hydroxy-2-methylpropiophenone).

##### Natural Wound Dressings

Natural lesion-covering hydrogels are seen as wound dressings that can promote wound healing with fewer adverse reactions. Dry skin wounds may be treated with natural hydrogels like Purilon^®^ Gel (sodium carboxymethylcellulose, calcium alginate) or ActivHeal Hydrogel (alginate), while ulcers and burns are treated with Prontosan wound gel (glycerol, hydroxyethyl cellulose, polyhexamethylene biguanide, undecylenamidopropyl betaine), Algicell Ag with antimicrobial silver alginate (calcium alginate with silver), or SoloSite Gel (sodium carboxymethylcellulose). The exudative lesions can be improved by Hyalofill (hyaluronic acid) when used as a wound dressing [261].

## 3. Conclusions

Chronic and acute wounds continue to pose major clinical and economic challenges, emphasizing the need for more effective wound care solutions. Hydrogels represent a promising class of wound dressings due to their biocompatibility, adaptability, and capacity to support the healing environment. Emerging hydrogel technologies, such as semisynthetic and stimuli-responsible hydrogels, offer enhanced functionality, including biocompatibility, antimicrobial action, tissue regeneration, drug delivery, and lower local pain. The strategic selection of appropriate in vitro and in vivo models remains critical to evaluating their therapeutic potential. While animal models have historically advanced our understanding of wound healing, their limitations highlight the growing importance of human in vitro skin models for preclinical testing. Continued innovation in hydrogel design and testing platforms holds great promise for improving patient outcomes, shortening healing times, and reducing the burden on healthcare systems.

## 4. Future Directions

The current research gaps in the development of hydrogels for wound healing are due to two main issues: developing specific wound-type hydrogels and biological evaluation.

In order to develop efficient hydrogels, there is a need for interdisciplinary collaboration between different specialists, such as chemists, physicians, biologists, and medical practitioners, to adapt the gels’ mechanical and chemical properties and biocompatibility to the biorequirements of each step of the healing process or wound type. These interactions will strongly influence five key cellular mechanisms, such as cell–cell communication, growth, migration, mechanic sensing, and tissue elaboration, reviewed in [262]. For instance, a modular hydrogel design, starting from synthetic polymers alone or combined with naturally derived ones to support biocompatibility, involves the specific formulation for a certain wound healing type; thus, it can be readily tested and translated in the clinic [263].

Hybrid hydrogels address these challenges by combining bioactive natural polymers with durable synthetic frameworks or responsive components. This enables the development of multifunctional dressings capable of adapting to the wound microenvironment, releasing antimicrobials, anti-inflammatories, or antioxidants, and promoting cell growth and differentiation. Their versatility, along with scalable potential and cost-effective production, makes hybrid and engineered synthetic hydrogels among the most promising platforms for clinical translation.

Additionally, computational modeling and artificial intelligence could help predict wound healing outcomes and optimize hydrogel formulations. Artificial intelligence (AI) and machine learning (ML) are increasingly recognized as transformative tools in hydrogel and wound dressing research, offering significant improvements over traditional trial-and-error approaches. Key properties of hydrogels, such as swelling behavior, mechanical strength, fluid handling, adhesion, tissue conformability, and biocompatibility, can be predicted with accuracy [264,265]. In wound care, AI-powered image analysis can automatically assess wound characteristics to guide material selection, predict healing outcomes, and personalize treatment strategies [266,267].

Future research in wound healing should prioritize the advancement of more accurate and standardized models that better mimic human physiology. A good assessment of hydrogels would require a standardized testing protocol that involves in vitro, in vivo, and human preclinical trials, eventually leading to clinical translation.

Existent in vitro and in vivo systems often fail to replicate the complexity of human wounds, particularly the immune response, vascularization, and local homeostatic imbalances leading to non-healing wounds. Given the limitations of animal models and increasing ethical concerns, there is a pressing need to adopt alternative testing strategies that reduce reliance on animal testing while still meeting regulatory standards.

Emerging technologies such as full-thickness skin, organ-on-a-chip, microfluidic platforms, and 3D bioprinted skin equivalents offer promise for the creation of dynamic, human-relevant models. These models can simulate critical wound conditions such as hypoxia, infection, and inflammation and enable more predictive testing of the hydrogel performance to facilitate a better understanding of their interaction with human tissues. With the advancing of personalized medicine care, tailored hydrogels, particularly stimuli-responsive types, designed to match a specific wound type and comorbidities (e.g., diabetes, vascular disease), will likely become a key focus. To support this, wound healing models will need to incorporate patient-derived cells and tissue samples to allow for personalized testing.

## Figures and Tables

**Table 1:** In vitro 2D models for wound healing studies.

Model Type	Characteristics	Advantages	Limitations	Typical Applications
Monolayer Culture [152,153,154]	single layer of cells on a flat surface	simple, cost-effective, and easy to analyze	limited cell–cell and ECM interactions	basic research, drug screening
Scratch Assay [152,155,156]	mechanical wound in monolayer	reproducible, widely used	not representative of tissue architecture	migration studies, wound closure assays
Transwell Assay [131,152,157]	migration through a porous membrane	quantitative, adaptable	no structural tissue context	chemotaxis, migration analysis
Co-culture System (2D) [127,131]	multiple cell types in a monolayer setup	intercellular communication, easy setup	hard to dissect specific pathways	interaction studies, signaling analysis

**Table 3:** Monitoring of the wound healing main processes on 2D and 3D in vitro models.

Process	2D Models	3D Models
Inflammation	inducible by pro-inflammatory stimuli assessment: inflammatory markers +: reproducible	layered cells with immune-like behavior assessment: inflammatory markers +: better simulate real inflammatory responses
Oxidative Stress	inducible by prooxidant stimuli	−: source identification of the marker, donor/time-dependent
Cell migration	assessment: imaging/oxidative stress markers +: reproducible	central hypoxia increases ROS +: reflects wound zone stress, assessment: oxidative stress markers −: source identification of the marker, donor/time dependent
Collagen Synthesis	scratch assay shows cell migration assessment: keratinocyte or fibroblast movement over time analyzed by imaging software, in dynamics	reconstructed skin models with epidermis with or without dermis −: difficult wound creation −: assessment by harvesting, HP imaging for re-epithelialization at different time points, and epithelial barrier function tests
Apoptosis	fibroblast cultures assessment: protein output/imaging	−: collagen deposited in scaffold or matrix assessment: imaging/biochemical assays

+ = advantages, − = disadvantages.

**Table 4:** In vivo surgical models used for experimental wound healing research.

No.	Model/Species	Treatment	Main Results
1	FTEW dorsal area—rabbits	Nanoemulsion hydrogel with Hypericum perforatum extract	Upregulated growth factors, increased vessel growth, and anti-microbial properties [172]
2	FTEW dorsal area—rats	Rutin-loaded zein gel	Promoted movement of cells towards the wound site, increased inflammation [173]
3	FTEW dorsal area—rats	Sodium thiosulfate gel	Increased fibroblast movement towards the wound bed, increased antioxidant enzymes [174]
4	FTEW dorsal area—mice	Gel containing *P. russeliana* extract	Low antioxidant effects, anti-inflammatory activity, promoted collagen formation, analgesic and pro-angiogenesis properties [175]
5	FTEW dorsal area—rats	Alginate hydrogel loaded with cerium oxide nanoparticles	Accelerated wound repair by reducing oxidative stress and inflammation, faster tissue recovery [169]
6	FTEW dorsal area—rats	Cream containing Astragalus floccosus extract	Accelerated wound healing by increased fibroblast proliferation, collagen synthesis, and re-epithelialization [176]
7	FTEW dorsal neck area—on Sprague Dawley rats	Sinomenine alkaloid solution extracted from Sinomenum Acutum	Faster wound closure with increased fibroblast proliferation and collagen deposition, antioxidant and anti-inflammatory effects [177]
8	FTEW (bilaterally punched dorsum wound) on C57BL/6 mice	mouse mesenchymal stem cells from hair follicles/dermal fibroblasts/growth factors	Reduced hypertrophic scarring and enhanced wound healing/combination therapy of MSCs, fibroblasts, and growth factors was the most effective [178]
9	FTEW (punched dorsum wound-6 mm) on rats	Sildenafil cream	Faster wound closure, reduced oxidative stress [179]
10	FTEW on rat and diabetic mouse, rat random flap, partial-thickness thermal injury on guinea pig	Systemic therapy with angiotensin (1–7)	Faster wound healing, increased cell proliferation without hypertensive effect, promoted regeneration, and flap survival [180]
11	SIW on rats and rabbits	Aloe ferox miller and Aloe Arborescens Miller whole leaf extracts	Faster healing, reduced inflammation, and antibacterial and antifungal activity with no skin toxicity [181]
12	FTEW dorsal area—BALB/c mice	Alchemilla vulgaris and Mimosa extract mixture	Rapid wound repair through improved skin re-epithelialization, higher cell proliferation, collagen synthesis, angiogenesis, and skin appendages [182]
13	FTEW dorsolateral flanks—Wistar rats	Ointment containing honey and Ageratum conyzoides leaf extract	Increased wound contraction, faster healing, and Ageratum extract reduced inflammation but increased fibrosis despite lower fibroblast count [183]
14	FTEW dorsal area—mice	chitosan patch with doxycycline/zinc/selenium nanoparticles	Anti-inflammatory and hemostatic effects, enhanced blood vessel formation [184]
15	Circular full-thickness skin excisions on a rat’s scalp	Chitosan membrane	Accelerated healing, anti-inflammatory effects by reduced leukocyte counts, and increased IL-4 levels [185]
16	FTEW dorsal area—diabetic Wistar rats	N-acetyl cysteine local and systemic	Reduced wound size and oxidative stress by both topical and systemic administration [186]
17	FTEW dorsal area—diabetic Wistar rats	Paeoniflorin-loaded hyaluronic acid hydrogel	Accelerated wound repair by anti-inflammatory effects mediated by modulation of macrophage populations (M1 towards M2 phenotype), enhanced angiogenesis, and collagen production [187]
18	FTEW dorsal area—nude mice	Heparinized adipose-derived scaffolds enriched with growth factors	Improved re-epithelialization, angiogenesis, and skin appendage regeneration through enhanced fibroblast migration and blood vessel growth [188]
19	FTEW dorsal area—mice	injection of mesenchymal Stem cell-derived extracellular vesicles/umbilical cord blood-derived extracellular vesicles in the wound margins	Enhanced wound healing by increased tissue growth and reduced scar formation [189]
20	FTEW dorsal area—rats	Neodymium–yttrium–aluminum garnet (Nd:YAG) pulsed high-intensity laser	Improved wound healing by increasing fibroblast proliferation, collagen synthesis, and thickness of the granular layer [190]
21	SI dorsal area—KO mice (double deletion of IL-10 and IL-4 genes)	Interleukin-10, local application	Improved wound healing, reduced inflammation, and enhanced scarring quality [191]
22	FTEW dorsal area—mice	Extracellular matrix/stromal vascular fraction gel conditioned medium	Faster wound closure, higher collagen deposition, increased growth factor secretion, and higher fibroblast and keratinocyte count and activity [192]
23	FTEW dorsal area—mice	Encapsulated Spirulina protein hydrolysates with nanoliposomes	Increased wound closure in mice by boosting fibroblast growth, skin regrowth, and enhanced markers of angiogenesis and collagen deposition [193]
24	FTEW dorsal area—mice	Silk protein-biomaterial wound dressings with epidermal growth factor and silver sulfadiazine	Better wound closure rate, less scarring, and collagen production [194]
25	FTEW dorsal area—C57 diabetic mice	Intermittent fasting and pulsed radiofrequency energy	Combined therapy exhibited antioxidant effects and induced faster wound healing by boosting cell migration, angiogenesis, and nerve growth [195]
26	Full-thickness SIW dorsal area—albino guinea pigs	Pulsed electrical stimulation	Both anodal and cathodal electrical stimulation boosted wound recovery in guinea pigs, improving closure and scar strength, regardless of polarity sequence [196]

FTEW = Full-thickness excisional wound, SIW = surgical incision wound.

**Table 2:** In vitro 3D models for wound healing studies.

Model Type	Characteristics	Advantages	Limitations	Typical Applications
3D Tissue Constructs [152]	multiple cell layers in the ECM	better tissue architecture, more realistic	higher complexity, longer setup	advanced research, tissue engineering
Co-culture System (3D) [155,156]	different cell types embedded in or layered within a 3D matrix	mimics natural skin structure and function	technically demanding	ECM remodeling, inflammatory studies
Hydrogel-based Models [30,131,158]	cells in/around gels like collagen or Matrigel	ECM simulation, tunable stiffness	batch variability, limited duration	ECM interaction, scaffold testing
Organotypic Culture [158]	stratified epidermis + fibroblast-populated dermis	closest to in vivo skin	time-consuming, resource-intensive	preclinical drug testing, epidermal healing
Explant Culture [159,160]	full-thickness skin cultured ex vivo	preserves native structure	short-term viability, donor variability	re-epithelialization, tissue remodeling
Microfluidic Skin-on-a-Chip [156,158]	skin model in a microfluidic device with a controlled environment	real-time imaging, dynamic flow conditions	expensive, specialized equipment needed	high-resolution studies, mechanistic modeling
Bioprinted Skin Constructs [161]	layered printing of cells and ECM components	reproducible, customizable architecture	high cost, needs specialized skills	personalized modeling, drug/cosmetic testing

## Data Availability

Data is contained within the article.
